# 1-[5-(3-Chloro­phen­yl)-2-methyl-3-thi­en­yl]-3,3,4,4,5,5-hexa­fluoro-2-(2-methoxy­phen­yl)­cyclo­pent-1-ene

**DOI:** 10.1107/S1600536809013993

**Published:** 2009-04-25

**Authors:** Congbin Fan, Weijun Liu, Gang Liu, Tianshe Yang

**Affiliations:** a Jiangxi Key Laboratory of Organic Chemistry, Jiangxi Science and Technology Normal University, Nanchang 330013, People’s Republic of China

## Abstract

The title compound, C_23_H_15_ClF_6_OS, has thienyl and phenyl­ene substituents on the double-bond C atoms of the envelope-shaped cyclo­pentenyl ring. The aromatic systems are aligned at 55.3 (4) (thien­yl) and 60.8 (7)° (phenyl­ene) with respect to the planar C—C=C—C portion of the main central cyclo­pentenyl ring.

## Related literature

For the synthesis of the precursors and related compounds, see: Fan *et al.* (2008 ▶, 2009 ▶); Pu *et al.* (2008 ▶).
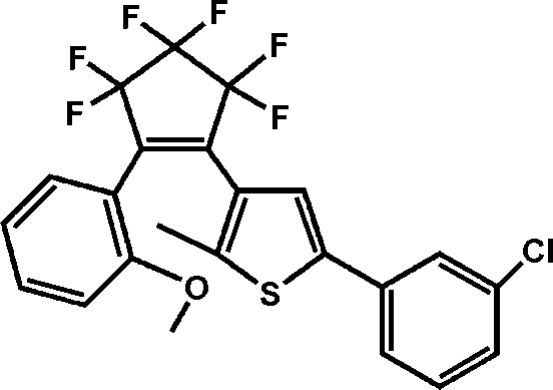


## Experimental

### 

#### Crystal data

C_23_H_15_ClF_6_OS
*M* *_r_* = 488.86
Triclinic, 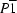

*a* = 9.4057 (10) Å
*b* = 10.2900 (11) Å
*c* = 11.9548 (13) Å
α = 83.529 (1)°
β = 71.564 (1)°
γ = 80.639 (1)°
*V* = 1080.7 (2) Å^3^
*Z* = 2
Mo *K*α radiation
μ = 0.34 mm^−1^
*T* = 291 K
0.49 × 0.45 × 0.33 mm

#### Data collection

Bruker SMART CCD area-detector diffractometer
Absorption correction: multi-scan (*SADABS*; Sheldrick, 1996 ▶) *T*_min_ = 0.851, *T*_max_ = 0.896
8254 measured reflections
3997 independent reflections
3358 reflections with *I* > 2σ(*I*)
*R*_int_ = 0.011

#### Refinement

*R*[*F* ^2^ > 2σ(*F* ^2^)] = 0.040
*wR*(*F* ^2^) = 0.111
*S* = 1.03
3997 reflections
321 parameters
553 restraints
H-atom parameters constrained
Δρ_max_ = 0.28 e Å^−3^
Δρ_min_ = −0.36 e Å^−3^

### 

Data collection: *SMART* (Bruker, 1997 ▶); cell refinement: *SAINT* (Bruker, 1997 ▶); data reduction: *SAINT*; program(s) used to solve structure: *SHELXS97* (Sheldrick, 2008 ▶); program(s) used to refine structure: *SHELXL97* (Sheldrick, 2008 ▶); molecular graphics: *ORTEPIII* (Burnett & Johnson, 1996 ▶) and *ORTEP-3 for Windows* (Farrugia, 1997 ▶); software used to prepare material for publication: *SHELXTL* (Sheldrick, 2008 ▶).

## Supplementary Material

Crystal structure: contains datablocks I, global. DOI: 10.1107/S1600536809013993/ng2571sup1.cif

Structure factors: contains datablocks I. DOI: 10.1107/S1600536809013993/ng2571Isup2.hkl

Additional supplementary materials:  crystallographic information; 3D view; checkCIF report

## Figures and Tables

**Fig. 1.**
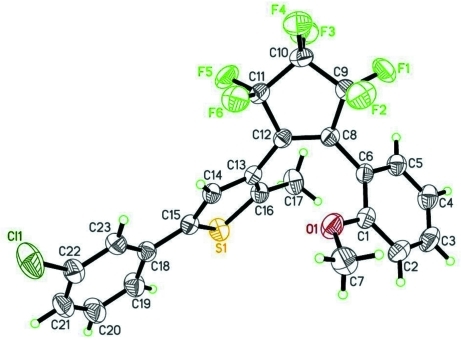
Molecular view the atom-labelling scheme. Ellipsoids are drawn at the 30% probability level. H atoms are represented as small spheres of arbitrary radii.

**Fig. 2.**
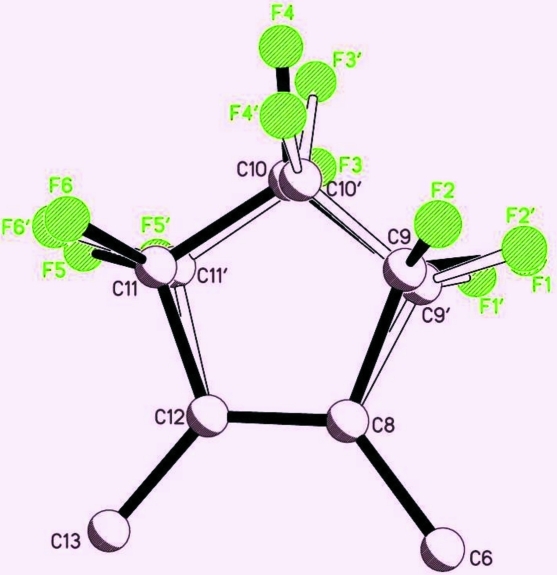
The diorder in cyclopent-1-ene ring.

## supplementary crystallographic information

### Comment

Crystal of the compound when dissolved in hexane can show photochromism. Upon irradiation with 365 nm light, the colorless crystals hexane solution turns blue rapidly. The blue compound displays an absorption maximum at 584 nm. Upon irradiation with visible light with a wavelength greater than 510 nm, the blue crystals hexane solution revert to their initial colorless state; a hexane solution has an absorption maximum at 291 nm. In a polymethylmethacylate amorphous film, the title diarylethene also demonstrates photochromism as similar as that in hexane.

### Experimental

The title compound was synthesized from the reaction of (2-methoxylpheny)perfluorocyclopent-1-ene (1.43 g, 4.75 mmol) (Fan *et al.*, 2009) and 3-bromo-2-methyl-5-(3-chlorophenyl)thiophene (1.44 g, 5 mmol) (Fan *et al.*, 2008) with *n*-butyllithium (2.0 ml, 5 mmol) at 195 K under a nitrogen atmosphere. After an hour, the reaction was quenced by the addition of water. The solid product was purified by column chromatography on silica with petroleum ether as the eluent to give the title compound 1.58 g (3.23 mmol) in 68% yield. C&H elemental analysis. Calc. for C_23_H_15_ClF_6_OS: C 56.51, H, 3.09%. Found C 55.92, H 3.15%.

### Refinement

All H atoms attached to C were fixed geometrically and treated as riding with C—H = 0.96 Å (methyl) or 0.93 Å (aromatic) with *U*_iso_(H) = 1.2*U*_eq_(aromatic) or *U*_iso_(H) = 1.5*U*_eq_(methyl). The cyclopent-1-ene ring in C9, C10, C11-envelope conformation is disorder, the C8—C9, C8—C9', C12—C11 and C12—C11' distance was strained to 1.50 (1) Å, respectively. The distance of C9—F1, C9—F2, F9'—F1', C9'—F2', C10—F3, C10—F4, C10'—F3', C10'—F4', C11—F5, C11—F6, C11'—F5'and C11—F6' was restrained to 1.34 (1) Å, respectively. The occupancies of the disorder components refined to a 0.784 (3):0.216 (3) ratio for C9:C9', F1:F1' and F2:F2'. The occupances of the disorder components refined to a 0.834 (5):0.166 (5) ratio for C11:C11', F5:F5', F6:F6' and 0.826 (2):0.174 (2) ratio for C10:C10', F3:F3', F4:F4'.

### Crystal data

**Table:**

C_23_H_15_ClF_6_OS	*Z* = 2
*M**_r_* = 488.86	*F*(000) = 496
Triclinic, *P*1	*D*_x_ = 1.502 Mg m^−^^3^
*a* = 9.4057 (10) Å	Mo *K*α radiation, λ = 0.71073 Å
*b* = 10.2900 (11) Å	Cell parameters from 4346 reflections
*c* = 11.9548 (13) Å	θ = 2.5–28.1°
α = 83.529 (1)°	µ = 0.34 mm^−^^1^
β = 71.564 (1)°	*T* = 291 K
γ = 80.639 (1)°	Block, colourless
*V* = 1080.7 (2) Å^3^	0.49 × 0.45 × 0.33 mm

### Data collection

**Table:**

Bruker SMART CCD area-detector diffractometer	3997 independent reflections
Radiation source: fine-focus sealed tube	3358 reflections with *I* > 2σ(*I*)
graphite	*R*_int_ = 0.011
φ and ω scans	θ_max_ = 25.5°, θ_min_ = 2.5°
Absorption correction: multi-scan (*SADABS*; Sheldrick, 1996)	*h* = −11→11
*T*_min_ = 0.851, *T*_max_ = 0.896	*k* = −12→12
8254 measured reflections	*l* = −14→14

### Refinement

**Table:**

Refinement on *F*^2^	Primary atom site location: structure-invariant direct methods
Least-squares matrix: full	Secondary atom site location: difference Fourier map
*R*[*F*^2^ > 2σ(*F*^2^)] = 0.040	Hydrogen site location: inferred from neighbouring sites
*wR*(*F*^2^) = 0.111	H-atom parameters constrained
*S* = 1.03	*w* = 1/[σ^2^(*F*_o_^2^) + (0.0497*P*)^2^ + 0.4496*P*] where *P* = (*F*_o_^2^ + 2*F*_c_^2^)/3
3997 reflections	(Δ/σ)_max_ = 0.001
321 parameters	Δρ_max_ = 0.28 e Å^−^^3^
553 restraints	Δρ_min_ = −0.36 e Å^−^^3^

### Special details

**Table:**

Geometry. All e.s.d.'s (except the e.s.d. in the dihedral angle between two l.s. planes) are estimated using the full covariance matrix. The cell e.s.d.'s are taken into account individually in the estimation of e.s.d.'s in distances, angles and torsion angles; correlations between e.s.d.'s in cell parameters are only used when they are defined by crystal symmetry. An approximate (isotropic) treatment of cell e.s.d.'s is used for estimating e.s.d.'s involving l.s. planes.
Refinement. Refinement of *F*^2^ against ALL reflections. The weighted *R*-factor *wR* and goodness of fit *S* are based on *F*^2^, conventional *R*-factors *R* are based on *F*, with *F* set to zero for negative *F*^2^. The threshold expression of *F*^2^ > σ(*F*^2^) is used only for calculating *R*-factors(gt) *etc*. and is not relevant to the choice of reflections for refinement. *R*-factors based on *F*^2^ are statistically about twice as large as those based on *F*, and *R*-factors based on ALL data will be even larger.

### Fractional atomic coordinates and isotropic or equivalent isotropic displacement parameters (Å^2^)

**Table:**

	*x*	*y*	*z*	*U*_iso_*/*U*_eq_	Occ. (<1)
C9	0.7086 (3)	0.7630 (3)	0.5031 (3)	0.0560 (8)	0.784 (3)
F1	0.5898 (3)	0.8609 (2)	0.5303 (2)	0.0930 (9)	0.784 (3)
F2	0.7750 (3)	0.7584 (2)	0.58991 (15)	0.0854 (7)	0.784 (3)
C9'	0.6784 (10)	0.7854 (12)	0.4846 (8)	0.0560 (8)	0.216 (3)
F1'	0.5498 (9)	0.8402 (8)	0.4640 (8)	0.0930 (9)	0.216 (3)
F2'	0.6890 (12)	0.8415 (10)	0.5755 (6)	0.0854 (7)	0.216 (3)
C10	0.6576 (3)	0.6302 (3)	0.5013 (3)	0.0566 (7)	0.826 (2)
F3	0.52853 (19)	0.6483 (2)	0.4715 (2)	0.0893 (6)	0.826 (2)
F4	0.6283 (3)	0.56317 (19)	0.60649 (15)	0.0877 (6)	0.826 (2)
C10'	0.6811 (14)	0.6391 (15)	0.5180 (12)	0.0566 (7)	0.174 (2)
F3'	0.5428 (9)	0.6053 (11)	0.5606 (12)	0.0893 (6)	0.174 (2)
F4'	0.7538 (10)	0.5982 (9)	0.5974 (7)	0.0877 (6)	0.174 (2)
C11	0.7876 (3)	0.5608 (3)	0.4040 (3)	0.0504 (6)	0.834 (5)
F5	0.7345 (3)	0.4861 (3)	0.34450 (17)	0.0778 (7)	0.834 (5)
F6	0.8815 (3)	0.4760 (2)	0.4529 (2)	0.0771 (7)	0.834 (5)
C11'	0.7621 (15)	0.5759 (12)	0.4012 (12)	0.0504 (6)	0.166 (5)
F5'	0.6680 (14)	0.5518 (13)	0.3457 (9)	0.0778 (7)	0.166 (5)
F6'	0.8458 (15)	0.4630 (13)	0.4176 (13)	0.0771 (7)	0.166 (5)
Cl1	1.52883 (10)	0.09741 (8)	0.13406 (7)	0.1001 (3)	
S1	1.10823 (7)	0.64743 (6)	−0.01005 (5)	0.05725 (18)	
O1	1.11475 (18)	0.81945 (15)	0.35023 (17)	0.0710 (5)	
C1	1.0232 (2)	0.9304 (2)	0.32810 (19)	0.0521 (5)	
C2	1.0714 (3)	1.0534 (2)	0.2928 (2)	0.0672 (6)	
H2	1.1715	1.0634	0.2819	0.081*	
C3	0.9705 (3)	1.1608 (2)	0.2740 (2)	0.0705 (7)	
H3	1.0039	1.2427	0.2500	0.085*	
C4	0.8232 (3)	1.1490 (2)	0.2899 (2)	0.0650 (6)	
H4	0.7562	1.2222	0.2777	0.078*	
C5	0.7744 (3)	1.0270 (2)	0.32442 (19)	0.0539 (5)	
H5	0.6738	1.0188	0.3356	0.065*	
C6	0.8730 (2)	0.91614 (18)	0.34282 (16)	0.0444 (4)	
C7	1.2362 (3)	0.8347 (3)	0.3900 (2)	0.0758 (7)	
H7A	1.3143	0.8689	0.3256	0.114*	
H7B	1.2755	0.7506	0.4198	0.114*	
H7C	1.2017	0.8949	0.4518	0.114*	
C8	0.8185 (2)	0.78631 (19)	0.38147 (17)	0.0442 (4)	
C12	0.8636 (2)	0.67244 (18)	0.32766 (16)	0.0413 (4)	
C13	0.9725 (2)	0.64498 (17)	0.21130 (16)	0.0419 (4)	
C14	1.0915 (2)	0.53693 (18)	0.19382 (17)	0.0440 (4)	
H14	1.1096	0.4798	0.2554	0.053*	
C15	1.1763 (2)	0.52489 (19)	0.07887 (17)	0.0461 (4)	
C16	0.9669 (2)	0.71461 (19)	0.10727 (18)	0.0488 (5)	
C17	0.8561 (3)	0.8294 (2)	0.0852 (2)	0.0666 (6)	
H17A	0.7603	0.8263	0.1451	0.100*	
H17B	0.8439	0.8248	0.0090	0.100*	
H17C	0.8932	0.9104	0.0873	0.100*	
C18	1.3086 (2)	0.4285 (2)	0.02687 (17)	0.0480 (4)	
C19	1.3943 (3)	0.4423 (2)	−0.0917 (2)	0.0658 (6)	
H19	1.3666	0.5128	−0.1399	0.079*	
C20	1.5198 (3)	0.3523 (3)	−0.1381 (2)	0.0772 (7)	
H20	1.5763	0.3638	−0.2170	0.093*	
C21	1.5622 (3)	0.2472 (3)	−0.0706 (2)	0.0681 (6)	
H21	1.6467	0.1868	−0.1025	0.082*	
C22	1.4773 (3)	0.2320 (2)	0.0462 (2)	0.0594 (5)	
C23	1.3525 (2)	0.3205 (2)	0.09609 (19)	0.0531 (5)	
H23	1.2977	0.3084	0.1754	0.064*	

### Atomic displacement parameters (Å^2^)

**Table:**

	*U*^11^	*U*^22^	*U*^33^	*U*^12^	*U*^13^	*U*^23^
C9	0.0574 (15)	0.0530 (16)	0.0522 (14)	−0.0082 (12)	−0.0075 (11)	−0.0065 (12)
F1	0.0854 (14)	0.0621 (11)	0.0886 (17)	0.0065 (10)	0.0275 (13)	−0.0103 (11)
F2	0.1147 (17)	0.1028 (17)	0.0470 (9)	−0.0445 (13)	−0.0213 (10)	−0.0054 (10)
C9'	0.0574 (15)	0.0530 (16)	0.0522 (14)	−0.0082 (12)	−0.0075 (11)	−0.0065 (12)
F1'	0.0854 (14)	0.0621 (11)	0.0886 (17)	0.0065 (10)	0.0275 (13)	−0.0103 (11)
F2'	0.1147 (17)	0.1028 (17)	0.0470 (9)	−0.0445 (13)	−0.0213 (10)	−0.0054 (10)
C10	0.0539 (14)	0.0575 (13)	0.0548 (14)	−0.0176 (11)	−0.0095 (10)	0.0055 (11)
F3	0.0540 (9)	0.0936 (14)	0.1218 (17)	−0.0148 (9)	−0.0273 (11)	−0.0044 (12)
F4	0.1098 (16)	0.0752 (11)	0.0590 (10)	−0.0282 (11)	0.0021 (10)	0.0153 (8)
C10'	0.0539 (14)	0.0575 (13)	0.0548 (14)	−0.0176 (11)	−0.0095 (10)	0.0055 (11)
F3'	0.0540 (9)	0.0936 (14)	0.1218 (17)	−0.0148 (9)	−0.0273 (11)	−0.0044 (12)
F4'	0.1098 (16)	0.0752 (11)	0.0590 (10)	−0.0282 (11)	0.0021 (10)	0.0153 (8)
C11	0.0549 (14)	0.0419 (12)	0.0553 (11)	−0.0108 (11)	−0.0182 (10)	0.0030 (9)
F5	0.0961 (16)	0.0636 (15)	0.0790 (10)	−0.0429 (13)	−0.0152 (10)	−0.0131 (10)
F6	0.0748 (13)	0.0608 (10)	0.0836 (16)	0.0013 (9)	−0.0233 (9)	0.0277 (10)
C11'	0.0549 (14)	0.0419 (12)	0.0553 (11)	−0.0108 (11)	−0.0182 (10)	0.0030 (9)
F5'	0.0961 (16)	0.0636 (15)	0.0790 (10)	−0.0429 (13)	−0.0152 (10)	−0.0131 (10)
F6'	0.0748 (13)	0.0608 (10)	0.0836 (16)	0.0013 (9)	−0.0233 (9)	0.0277 (10)
Cl1	0.0996 (6)	0.0937 (5)	0.0895 (5)	0.0389 (4)	−0.0325 (4)	0.0023 (4)
S1	0.0699 (4)	0.0557 (3)	0.0415 (3)	0.0014 (3)	−0.0171 (2)	0.0026 (2)
O1	0.0581 (9)	0.0508 (9)	0.1132 (14)	−0.0043 (7)	−0.0380 (9)	−0.0118 (9)
C1	0.0571 (12)	0.0424 (10)	0.0589 (12)	−0.0093 (9)	−0.0176 (10)	−0.0084 (9)
C2	0.0677 (14)	0.0549 (13)	0.0837 (16)	−0.0232 (11)	−0.0220 (12)	−0.0052 (12)
C3	0.0964 (19)	0.0432 (12)	0.0748 (16)	−0.0224 (12)	−0.0259 (14)	0.0020 (11)
C4	0.0873 (17)	0.0440 (12)	0.0642 (14)	−0.0038 (11)	−0.0281 (12)	0.0024 (10)
C5	0.0603 (12)	0.0479 (11)	0.0533 (12)	−0.0045 (9)	−0.0188 (10)	−0.0024 (9)
C6	0.0521 (11)	0.0388 (10)	0.0421 (10)	−0.0067 (8)	−0.0127 (8)	−0.0059 (8)
C7	0.0712 (16)	0.0849 (18)	0.0764 (17)	−0.0021 (14)	−0.0305 (13)	−0.0147 (14)
C8	0.0458 (10)	0.0430 (10)	0.0450 (10)	−0.0086 (8)	−0.0144 (8)	−0.0029 (8)
C12	0.0436 (10)	0.0388 (9)	0.0451 (10)	−0.0074 (8)	−0.0181 (8)	−0.0002 (8)
C13	0.0484 (10)	0.0366 (9)	0.0436 (10)	−0.0088 (8)	−0.0171 (8)	−0.0016 (7)
C14	0.0510 (11)	0.0389 (9)	0.0429 (10)	−0.0050 (8)	−0.0171 (8)	0.0006 (8)
C15	0.0516 (11)	0.0424 (10)	0.0457 (10)	−0.0070 (8)	−0.0169 (9)	−0.0019 (8)
C16	0.0577 (12)	0.0431 (10)	0.0471 (11)	−0.0033 (9)	−0.0203 (9)	−0.0015 (8)
C17	0.0837 (17)	0.0573 (13)	0.0593 (13)	0.0119 (12)	−0.0337 (12)	−0.0014 (11)
C18	0.0510 (11)	0.0471 (11)	0.0461 (10)	−0.0060 (8)	−0.0142 (9)	−0.0067 (8)
C19	0.0787 (16)	0.0590 (13)	0.0490 (12)	−0.0009 (11)	−0.0087 (11)	−0.0029 (10)
C20	0.0825 (17)	0.0754 (17)	0.0548 (14)	−0.0005 (14)	0.0032 (12)	−0.0114 (12)
C21	0.0601 (14)	0.0703 (15)	0.0658 (15)	0.0051 (11)	−0.0086 (11)	−0.0218 (12)
C22	0.0572 (13)	0.0586 (13)	0.0627 (13)	0.0036 (10)	−0.0226 (11)	−0.0089 (10)
C23	0.0513 (11)	0.0568 (12)	0.0477 (11)	−0.0030 (9)	−0.0115 (9)	−0.0058 (9)

### Geometric parameters (Å, °)

**Table:**

C9—F1	1.359 (3)	C3—H3	0.9300
C9—F2	1.363 (4)	C4—C5	1.381 (3)
C9—C8	1.514 (3)	C4—H4	0.9300
C9—C10	1.525 (4)	C5—C6	1.392 (3)
C9'—F2'	1.324 (9)	C5—H5	0.9300
C9'—F1'	1.332 (9)	C6—C8	1.481 (3)
C9'—C8	1.493 (8)	C7—H7A	0.9600
C9'—C10'	1.512 (9)	C7—H7B	0.9600
C10—F4	1.334 (3)	C7—H7C	0.9600
C10—F3	1.349 (3)	C8—C12	1.342 (3)
C10—C11	1.536 (4)	C12—C13	1.469 (3)
C10'—F3'	1.327 (10)	C13—C16	1.375 (3)
C10'—F4'	1.332 (10)	C13—C14	1.426 (3)
C10'—C11'	1.524 (9)	C14—C15	1.361 (3)
C11—F5	1.348 (3)	C14—H14	0.9300
C11—F6	1.358 (3)	C15—C18	1.472 (3)
C11—C12	1.510 (3)	C16—C17	1.502 (3)
C11'—F6'	1.326 (10)	C17—H17A	0.9600
C11'—F5'	1.328 (10)	C17—H17B	0.9600
C11'—C12	1.500 (9)	C17—H17C	0.9600
Cl1—C22	1.741 (2)	C18—C19	1.396 (3)
S1—C16	1.718 (2)	C18—C23	1.396 (3)
S1—C15	1.731 (2)	C19—C20	1.380 (3)
O1—C1	1.368 (3)	C19—H19	0.9300
O1—C7	1.405 (3)	C20—C21	1.359 (4)
C1—C2	1.389 (3)	C20—H20	0.9300
C1—C6	1.398 (3)	C21—C22	1.377 (3)
C2—C3	1.383 (4)	C21—H21	0.9300
C2—H2	0.9300	C22—C23	1.376 (3)
C3—C4	1.361 (4)	C23—H23	0.9300
			
F1—C9—F2	105.1 (3)	C5—C6—C8	120.66 (18)
F1—C9—C8	112.8 (2)	C1—C6—C8	120.53 (17)
F2—C9—C8	111.9 (2)	O1—C7—H7A	109.5
F1—C9—C10	111.9 (3)	O1—C7—H7B	109.5
F2—C9—C10	110.1 (2)	H7A—C7—H7B	109.5
C8—C9—C10	105.2 (2)	O1—C7—H7C	109.5
F2'—C9'—F1'	107.7 (8)	H7A—C7—H7C	109.5
F2'—C9'—C8	112.1 (8)	H7B—C7—H7C	109.5
F1'—C9'—C8	116.1 (8)	C12—C8—C6	129.26 (18)
F2'—C9'—C10'	107.2 (10)	C12—C8—C9'	114.1 (5)
F1'—C9'—C10'	112.4 (9)	C6—C8—C9'	115.9 (5)
C8—C9'—C10'	100.9 (7)	C12—C8—C9	109.6 (2)
F4—C10—F3	106.5 (2)	C6—C8—C9	120.8 (2)
F4—C10—C9	112.6 (3)	C8—C12—C13	129.51 (17)
F3—C10—C9	110.1 (3)	C8—C12—C11'	106.6 (6)
F4—C10—C11	114.1 (3)	C13—C12—C11'	123.3 (6)
F3—C10—C11	110.1 (2)	C8—C12—C11	111.5 (2)
C9—C10—C11	103.5 (2)	C13—C12—C11	118.97 (19)
F3'—C10'—F4'	108.1 (9)	C16—C13—C14	112.60 (17)
F3'—C10'—C9'	111.9 (11)	C16—C13—C12	124.45 (17)
F4'—C10'—C9'	112.7 (11)	C14—C13—C12	122.85 (16)
F3'—C10'—C11'	109.3 (11)	C15—C14—C13	113.91 (17)
F4'—C10'—C11'	111.1 (11)	C15—C14—H14	123.0
C9'—C10'—C11'	103.7 (9)	C13—C14—H14	123.0
F5—C11—F6	105.7 (2)	C14—C15—C18	129.71 (18)
F5—C11—C12	113.2 (2)	C14—C15—S1	109.86 (15)
F6—C11—C12	112.9 (2)	C18—C15—S1	120.42 (15)
F5—C11—C10	110.8 (2)	C13—C16—C17	129.94 (19)
F6—C11—C10	110.0 (3)	C13—C16—S1	110.41 (15)
C12—C11—C10	104.2 (2)	C17—C16—S1	119.58 (16)
F6'—C11'—F5'	107.9 (9)	C16—C17—H17A	109.5
F6'—C11'—C12	109.1 (11)	C16—C17—H17B	109.5
F5'—C11'—C12	110.4 (9)	H17A—C17—H17B	109.5
F6'—C11'—C10'	110.9 (12)	C16—C17—H17C	109.5
F5'—C11'—C10'	113.1 (11)	H17A—C17—H17C	109.5
C12—C11'—C10'	105.4 (8)	H17B—C17—H17C	109.5
C16—S1—C15	93.21 (9)	C19—C18—C23	118.1 (2)
C1—O1—C7	118.22 (18)	C19—C18—C15	121.50 (19)
O1—C1—C2	123.8 (2)	C23—C18—C15	120.39 (18)
O1—C1—C6	116.66 (17)	C20—C19—C18	120.6 (2)
C2—C1—C6	119.5 (2)	C20—C19—H19	119.7
C3—C2—C1	120.0 (2)	C18—C19—H19	119.7
C3—C2—H2	120.0	C21—C20—C19	121.2 (2)
C1—C2—H2	120.0	C21—C20—H20	119.4
C4—C3—C2	121.2 (2)	C19—C20—H20	119.4
C4—C3—H3	119.4	C20—C21—C22	118.6 (2)
C2—C3—H3	119.4	C20—C21—H21	120.7
C3—C4—C5	119.2 (2)	C22—C21—H21	120.7
C3—C4—H4	120.4	C23—C22—C21	122.0 (2)
C5—C4—H4	120.4	C23—C22—Cl1	118.70 (18)
C4—C5—C6	121.3 (2)	C21—C22—Cl1	119.34 (18)
C4—C5—H5	119.4	C22—C23—C18	119.6 (2)
C6—C5—H5	119.4	C22—C23—H23	120.2
C5—C6—C1	118.77 (18)	C18—C23—H23	120.2
			
F1—C9—C10—F4	−89.8 (3)	C10—C9—C8—C12	−16.4 (3)
F2—C9—C10—F4	26.7 (4)	F1—C9—C8—C6	47.6 (4)
C8—C9—C10—F4	147.5 (3)	F2—C9—C8—C6	−70.7 (3)
F1—C9—C10—F3	28.9 (4)	C10—C9—C8—C6	169.7 (2)
F2—C9—C10—F3	145.4 (3)	F1—C9—C8—C9'	−29.7 (18)
C8—C9—C10—F3	−93.8 (3)	F2—C9—C8—C9'	−148 (2)
F1—C9—C10—C11	146.6 (3)	C10—C9—C8—C9'	92 (2)
F2—C9—C10—C11	−96.9 (3)	C6—C8—C12—C13	−4.6 (3)
C8—C9—C10—C11	23.8 (3)	C9'—C8—C12—C13	165.0 (5)
F2'—C9'—C10'—F3'	95.5 (11)	C9—C8—C12—C13	−177.7 (2)
F1'—C9'—C10'—F3'	−22.6 (13)	C6—C8—C12—C11'	−175.7 (6)
C8—C9'—C10'—F3'	−147.0 (10)	C9'—C8—C12—C11'	−6.1 (7)
F2'—C9'—C10'—F4'	−26.6 (13)	C9—C8—C12—C11'	11.2 (6)
F1'—C9'—C10'—F4'	−144.7 (9)	C6—C8—C12—C11	174.6 (2)
C8—C9'—C10'—F4'	90.9 (11)	C9'—C8—C12—C11	−15.8 (5)
F2'—C9'—C10'—C11'	−146.8 (9)	C9—C8—C12—C11	1.5 (3)
F1'—C9'—C10'—C11'	95.0 (10)	F6'—C11'—C12—C8	−132.9 (9)
C8—C9'—C10'—C11'	−29.3 (10)	F5'—C11'—C12—C8	108.7 (10)
F4—C10—C11—F5	92.5 (3)	C10'—C11'—C12—C8	−13.7 (10)
F3—C10—C11—F5	−27.2 (3)	F6'—C11'—C12—C13	55.3 (12)
C9—C10—C11—F5	−144.9 (2)	F5'—C11'—C12—C13	−63.1 (12)
F4—C10—C11—F6	−24.1 (3)	C10'—C11'—C12—C13	174.5 (6)
F3—C10—C11—F6	−143.8 (3)	F6'—C11'—C12—C11	−13 (3)
C9—C10—C11—F6	98.5 (3)	F5'—C11'—C12—C11	−131 (4)
F4—C10—C11—C12	−145.4 (2)	C10'—C11'—C12—C11	106 (4)
F3—C10—C11—C12	94.9 (3)	F5—C11—C12—C8	134.4 (2)
C9—C10—C11—C12	−22.8 (3)	F6—C11—C12—C8	−105.5 (3)
F3'—C10'—C11'—F6'	−95.1 (13)	C10—C11—C12—C8	13.9 (3)
F4'—C10'—C11'—F6'	24.1 (13)	F5—C11—C12—C13	−46.3 (3)
C9'—C10'—C11'—F6'	145.4 (10)	F6—C11—C12—C13	73.8 (3)
F3'—C10'—C11'—F5'	26.3 (13)	C10—C11—C12—C13	−166.80 (19)
F4'—C10'—C11'—F5'	145.5 (10)	F5—C11—C12—C11'	71 (4)
C9'—C10'—C11'—F5'	−93.2 (10)	F6—C11—C12—C11'	−169 (4)
F3'—C10'—C11'—C12	146.9 (10)	C10—C11—C12—C11'	−49 (4)
F4'—C10'—C11'—C12	−93.8 (11)	C8—C12—C13—C16	−52.8 (3)
C9'—C10'—C11'—C12	27.4 (12)	C11'—C12—C13—C16	117.0 (7)
C7—O1—C1—C2	28.8 (3)	C11—C12—C13—C16	128.1 (2)
C7—O1—C1—C6	−151.1 (2)	C8—C12—C13—C14	131.0 (2)
O1—C1—C2—C3	−179.1 (2)	C11'—C12—C13—C14	−59.2 (7)
C6—C1—C2—C3	0.8 (4)	C11—C12—C13—C14	−48.2 (3)
C1—C2—C3—C4	0.3 (4)	C16—C13—C14—C15	0.6 (2)
C2—C3—C4—C5	−0.6 (4)	C12—C13—C14—C15	177.27 (17)
C3—C4—C5—C6	−0.2 (3)	C13—C14—C15—C18	178.83 (18)
C4—C5—C6—C1	1.3 (3)	C13—C14—C15—S1	−0.5 (2)
C4—C5—C6—C8	179.1 (2)	C16—S1—C15—C14	0.21 (16)
O1—C1—C6—C5	178.30 (19)	C16—S1—C15—C18	−179.19 (16)
C2—C1—C6—C5	−1.6 (3)	C14—C13—C16—C17	176.5 (2)
O1—C1—C6—C8	0.5 (3)	C12—C13—C16—C17	−0.1 (3)
C2—C1—C6—C8	−179.4 (2)	C14—C13—C16—S1	−0.4 (2)
C5—C6—C8—C12	120.1 (2)	C12—C13—C16—S1	−177.03 (14)
C1—C6—C8—C12	−62.1 (3)	C15—S1—C16—C13	0.14 (16)
C5—C6—C8—C9'	−49.3 (5)	C15—S1—C16—C17	−177.15 (18)
C1—C6—C8—C9'	128.5 (5)	C14—C15—C18—C19	−171.0 (2)
C5—C6—C8—C9	−67.4 (3)	S1—C15—C18—C19	8.3 (3)
C1—C6—C8—C9	110.4 (2)	C14—C15—C18—C23	8.5 (3)
F2'—C9'—C8—C12	137.0 (8)	S1—C15—C18—C23	−172.26 (16)
F1'—C9'—C8—C12	−98.6 (9)	C23—C18—C19—C20	−0.8 (4)
C10'—C9'—C8—C12	23.2 (8)	C15—C18—C19—C20	178.7 (2)
F2'—C9'—C8—C6	−52.0 (10)	C18—C19—C20—C21	0.9 (4)
F1'—C9'—C8—C6	72.5 (10)	C19—C20—C21—C22	−0.2 (4)
C10'—C9'—C8—C6	−165.8 (6)	C20—C21—C22—C23	−0.6 (4)
F2'—C9'—C8—C9	59.4 (16)	C20—C21—C22—Cl1	179.5 (2)
F1'—C9'—C8—C9	−176 (3)	C21—C22—C23—C18	0.6 (3)
C10'—C9'—C8—C9	−54.4 (18)	Cl1—C22—C23—C18	−179.50 (17)
F1—C9—C8—C12	−138.6 (3)	C19—C18—C23—C22	0.1 (3)
F2—C9—C8—C12	103.1 (3)	C15—C18—C23—C22	−179.40 (19)
